# Practical guidelines on imaging of retinoblastoma: a 2025 update on behalf of the European Retinoblastoma Imaging Collaboration and the European Retinoblastoma Group

**DOI:** 10.1007/s00330-025-11853-1

**Published:** 2025-08-06

**Authors:** Liesbeth Cardoen, Selma Sirin, Paolo Galluzzi, Marcus C. de Jong, Meriam Koob, Sophia Göricke, Christiaan M. de Bloeme, Maaike Moor, Isabelle Aerts, Alexandre Matet, Annette C. Moll, François Doz, Pim de Graaf, Hervé J. Brisse

**Affiliations:** 1 Imaging Department, Institut Curie, Université Paris Sciences et Lettres, Paris, France; 2 Department of Diagnostic Imaging, University Children’s Hospital Zürich, Zürich, Switzerland; 3 Neuroradiology Unit Azienda Ospedaliera Universitaria Santa Maria alle Scotte, Siena, Italy; 4 Department of Radiology and Nuclear Medicine, Amsterdam UMC location Vrije Universiteit Amsterdam, Cancer Center Amsterdam, Imaging and Biomarkers, Amsterdam, The Netherlands; 5 Department of Radiology, Lausanne University Hospital (CHUV) and University of Lausanne (UNIL), Lausanne, Switzerland; 6 Institute of Diagnostic and Interventional Radiology and Neuroradiology, University Hospital Essen, Essen, Germany; 7 SIREDO Oncology Center Care, Innovation and Research for Children, Adolescents and Young Adults with Cancer, Institut Curie, Paris, France; 8 Department of Ophthalmology, Institut Curie, Université Paris Cité, Paris, France; 9 Department of Ophthalmology, Amsterdam UMC location Vrije Universiteit Amsterdam, Cancer Center Amsterdam, Cancer Treatment and Quality of Life, Amsterdam, The Netherlands

**Keywords:** Retinoblastoma, Eye neoplasms, Magnetic resonance imaging, Ultrasonography, ocular, Guidelines as topic

## Abstract

**Abstract:**

Retinoblastoma is the most common intraocular malignancy in children. Imaging plays a crucial role in evaluating children with retinoblastoma, both to determine tumor extension and to confirm the diagnosis when necessary. The eighth edition of the American Joint Committee on Cancer (TNM) classification provides a comprehensive intraocular and extraocular staging for retinoblastoma, with a key role for imaging, particularly in cases of advanced tumors. Imaging guidelines for retinoblastoma were first outlined by the European Retinoblastoma Imaging Collaboration (ERIC) in 2012. Over the past decade, technological advancements have enabled the acquisition of higher-resolution MR images and expanded our understanding of clinically significant imaging features of retinoblastoma. This manuscript aims to highlight these evolving insights in retinoblastoma imaging and to promote standardization of imaging practices. We propose an updated MR protocol and a standardized reporting system based on the key points essential for managing patients with retinoblastoma by ophthalmologists and pediatric oncologists.

**Key Points:**

***Question*** *Advancements in imaging technology and updated TNM classification require revising retinoblastoma imaging guidelines to standardize practices and improve accuracy.*

***Findings*** *High-spatial-resolution contrast-enhanced MRI, performed within two weeks of diagnosis, under general anesthesia, is essential for pretreatment staging. The MRI protocol must include mandatory sequences for accuracy.*

***Clinical relevance*** *Retinoblastoma imaging is essential for diagnosis and tumor extension assessment. The updated guidelines, aligned with the eighth edition of the AJCC TNM classification, aim to standardize practices, improve imaging accuracy, and assist ophthalmologists and pediatric oncologists in patient management.*

## Introduction

Retinoblastoma is the most common intraocular malignancy in children. The ophthalmologist usually diagnoses retinoblastoma through fundoscopy and ultrasonography while the patient is under general anesthesia. Imaging is crucial not only to confirm the diagnosis when fundoscopy is limited due to opaque media, but also to differentiate retinoblastoma from other conditions that may mimic it. Once retinoblastoma is diagnosed, MRI is essential for assessing tumor extension.

Currently, the eighth edition of the American Joint Committee on Cancer (TNM) classification (Table 1) provides a comprehensive intraocular and extraocular staging of retinoblastoma [1].

**Table 1:**
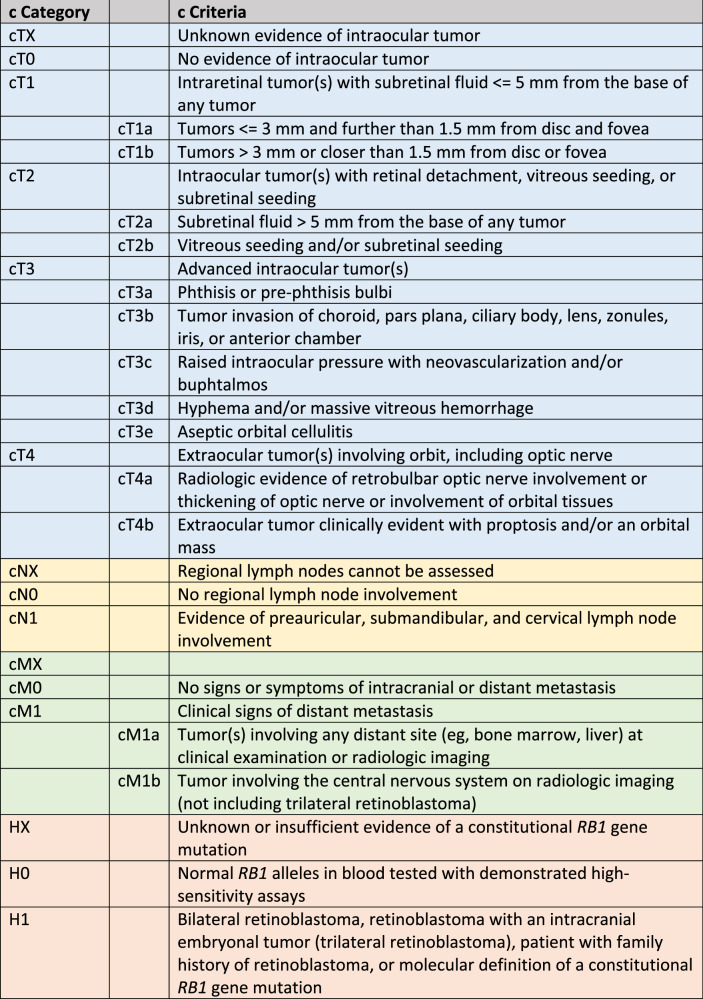
The eighth edition of the American Joint Committee on Cancer TNM clinical classification for retinoblastoma [1]

Imaging guidelines for retinoblastoma were first introduced by the ERIC in 2012 [2] and have since been adapted [3]. Over the past decade, advances in imaging technology have enabled higher spatial resolution in MRI. Additionally, research [4–30] has expanded our understanding of relevant imaging features, such as the assessment of tumor invasion into the optic nerve or the presence of trilateral retinoblastoma, while also refining criteria to more accurately differentiate between retinoblastoma and non-tumoral conditions.

To develop updated imaging guidelines, a structured Delphi process was employed, incorporating recent literature and expert input through multiple iterative discussion rounds.

The aim of this manuscript is to present these evolving insights in retinoblastoma imaging and to promote further standardization of MRI protocols and reporting.

## Imaging modalities and techniques

### Ultrasound imaging

Ultrasound imaging can complement fundoscopy in diagnosing retinoblastoma [31], especially when complications like retinal detachment, intraocular hemorrhage or cataract interfere with fundoscopy. The presence of microcalcifications in an ocular mass in young children is nearly pathognomonic for retinoblastoma [32]. In cases of diagnostic uncertainty, ultrasonography is the first-line imaging modality for confirming intratumoral calcifications.

### CT

**Fig. 1:**
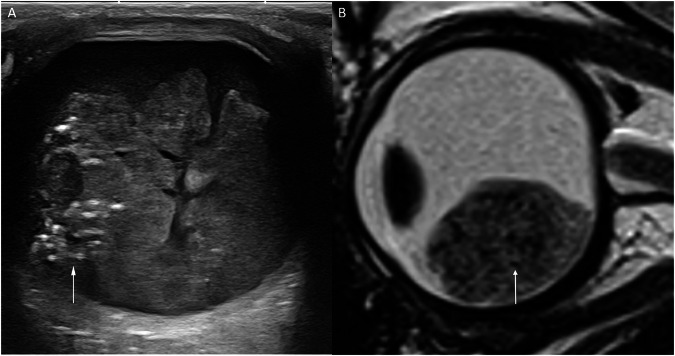
Microcalcifications in retinoblastoma: **A** Ultrasound image obtained using a high-frequency (up to 22 MHz) linear probe, showing a heterogeneous hyperechoic intraocular mass arising from the retina, with highly reflective shadowing (arrow) suggestive of calcification, and nearly pathognomonic for retinoblastoma. **B** Sagittal T2-weighted spin echo MR image revealing a solid intraocular mass containing multiple hypointense spots, compatible with microcalcifications

CT is no longer recommended for retinoblastoma evaluation. Ultrasonography, along with high-resolution spin echo T2 or susceptibility-weighted MRI, is sufficient to assess intratumoral calcifications (Fig. 1) [5]. If MRI is available, it should be preferred over CT due to CT’s low sensitivity and specificity in detecting locoregional extension [6, 33, 34], as well as its radiation exposure risk, which is particularly concerning for children with tumor-predisposing genetic conditions.

### MRI

High-resolution contrast-enhanced MRI is essential for pretreatment retinoblastoma staging, assessing intra- and extraocular spread, and identifying potential intracranial involvement.

#### Hardware considerations

**Fig. 2:**
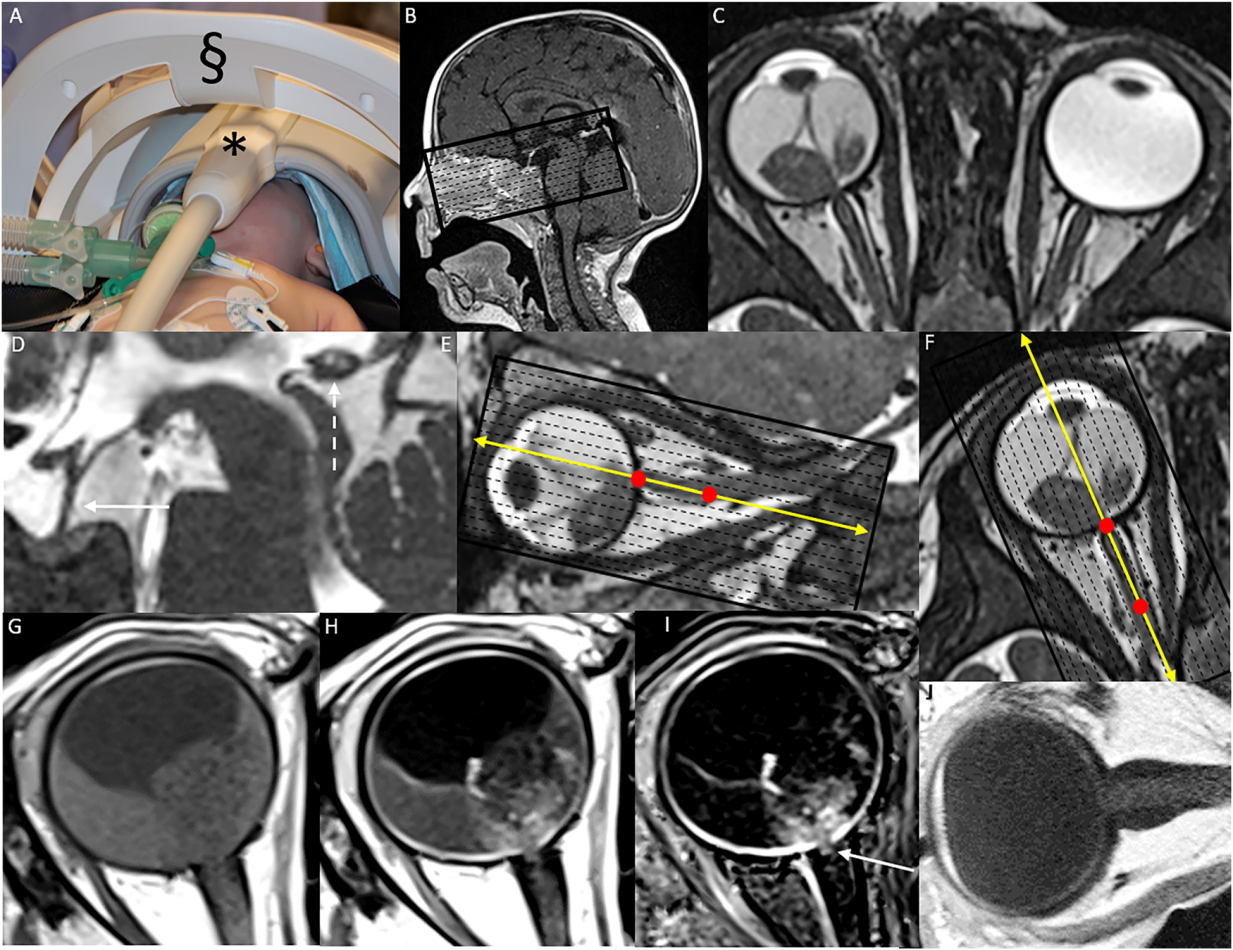
Placement of an infant in an MRI machine and 3D heavily T2-weighted sequence and 2D T1-weighted SE images centered on the orbits: **A** The infant, under general anesthesia, is positioned with an orbit surface coil (asterisk) and a head coil (section) for imaging in a 1.5-T MRI machine. **B** A scout image shows the selected area of interest, centered on the orbits and including the pineal region. **C** Axial heavily T2-weighted MR image, centered on the orbits and oriented in the plane of the optic nerve, revealing an exophytic intraocular mass in the right eye, associated with complete retinal detachment. **D** Sagittal reconstruction showing a normal suprasellar region (arrow) and a normal pineal gland (dashed arrow). **E** The axial and **F** sagittal obliques T1 sequences are planned along the orientation of the retrobulbar portion of the optic nerve (red dots), based on a reconstructed sagittal and axial plane of the 3D high-resolution heavily T2-weighted images. The central slice must be positioned precisely in the middle of the nerve (yellow arrows). **G** Axial T1-weighted sequence before contrast and **H** axial T1-weighted sequence after contrast, both oriented along the optic nerve axis. **I** Subtraction of the pre- and post-contrast T1-weighted sequences reveals subtle contrast enhancement (arrow), indicating postlaminar optic nerve infiltration. Although Dixon sequences can also be used, they have the drawback of increased acquisition time due to fat saturation. **J** Sagittal T1-weighted sequence after contrast, oriented along the optic nerve axis

For optimal imaging quality, images should be acquired on a 3-T system with a multi-channel head coil [7], or on a 1.5-T system with surface coils ideally covering both eyes. The head and surface coils can be positioned in parallel but should remain uncoupled during acquisition (Fig. 2A).

#### Sedation

General anesthesia is essential for obtaining high-quality, high-spatial-resolution MR images as it minimizes eye movement, which is crucial even with effective contention methods. If general anesthesia is not feasible in newborns or infants under 6 months, MRI may be performed using the “feed-and-swaddle” technique, which involves sleep deprivation, followed by feeding and tightly swaddling the infant before positioning them in the MRI machine. In such cases, a head coil may be used, and the baby’s head should be elevated and positioned as close as possible to the coil. However, this technique carries the risk of suboptimal high-resolution image quality, particularly of the optic nerve. Therefore, it is not recommended. If necessary, the MRI should be repeated under appropriate conditions.

#### Optimized pulse sequences and high spatial resolution

A comprehensive MRI protocol for retinoblastoma should include high-resolution imaging of the orbit with an in-plane pixel size of ≤ 0.3 × 0.3 mm and a slice thickness of ≤ 2 mm, as well as standard-resolution imaging of the entire brain.

##### MR Imaging of the orbit.

An axial isotropic 3D heavily T2-weighted sequence with a slice thickness of ≤ 0.5 mm provides high-resolution images of both orbits and eyes, enabling the detection of small intraocular tumor foci and seeds. Preferably, this sequence should also include the pineal gland, or alternatively, a sagittal T2-weighted spin echo (SE) sequence can be used to evaluate the pineal and suprasellar regions (Fig. 2B–D).

Pre- and post-contrast 2D axial T1-weighted SE images are crucial for detecting invasion of intraocular and extraocular structures. It is critical to align the orientation of these slices with the most distal (retrobulbar) segment of the optic nerve, ensuring that the central slice is positioned precisely in the middle of the nerve (Fig. 2E). This significantly enhances diagnostic accuracy in detecting early-stage optic nerve invasion. Fat suppression may improve visualization of enhancement in the optic nerve, meningeal sheaths, extraocular disease, and orbital inflammatory changes (Fig. 2I). However, non-fat-suppressed sequences may provide better spatial resolution, sensitivity, and specificity for detecting choroidal invasion (Fig. 2H) [8]. Depending on institutional preference and time constraints, two approaches are proposed. The acquisition of 2D T1 Spin Echo sequences without fat saturation before and after contrast injection must be followed by a subtraction technique. Alternatively, sequences with and without fat saturation—such as DIXON—can be acquired in a single acquisition, which also allows for the generation of subtraction images. It is noted that acquisition times for fat-saturated sequences are generally longer than those without fat saturation, which may influence protocol selection in time-limited clinical settings.

Oblique-sagittal 2D contrast-enhanced T1-weighted images, centered on the optic nerve axis, are essential for evaluating the choroid and optic nerve (Fig. 2F, J).

Axial multi-shot or non-EPI DWI of the orbit, with a slice thickness of < 4 mm, is necessary for assessing tumor presence within or beyond the eye and may aid in the differential diagnosis of non-tumoral conditions [28, 35].

Axial or sagittal T2-weighted SE images centered on the orbits offer high contrast, aiding in the identification of intralesional calcifications and in assessing the signal characteristics of the optic nerve.

Susceptibility-weighted sequences can confirm intralesional microcalcifications but are only necessary when the diagnosis is uncertain, as fundoscopy with ultrasonography usually diagnoses retinoblastoma before MRI.

##### MR Imaging of the brain and spinal axis.

A contrast-enhanced 3D T1-weighted SE sequence covering the entire brain allows a comprehensive analysis, focusing on the pineal and suprasellar region, the leptomeninges and potential brain anomalies.

Optional sequences include DWI, T2 SE or T2 FLAIR sequences of the brain. A contrast-enhanced T2 Flair sequence can improve the detection of leptomeningeal dissemination [36].

If intra-arterial chemotherapy is being considered, high-resolution 3D heavily T2-weighted sequences or an additional TOF sequence provide valuable information about the anatomical origin of the ophthalmic artery for vascular access.

In cases of extensive optic nerve invasion, meningeal sheath extension, or suspicion of a midline embryonal tumor, imaging of the entire spinal axis, including the dural sac, is mandatory to assess for spinal leptomeningeal spread.

The complete retinoblastoma MRI protocol is summarized in Table 2.

**Table 2:** MRI protocol for retinoblastoma with key points for analysis

Sequences	Plane	Slice thickness	In-plane pixel size	Imaging analysis
Mandatory				
3D high-resolution heavily T2^a^	Axial	≤ 0.5 mm	≤ 0.3 × 0.3 mm	Number, size and location of tumors Retinal detachment Vitreous and subretinal seeds Size and signal of the optic nerve Pineal gland/suprasellar region Origin and course of the ophthalmic artery
2D T1 Spin Echo^a,b^ or DIXON^a^	Oblique axial	≤ 2 mm	≤ 0.3 × 0.3 mm	Detection of bleeding/protein-rich subretinal fluid
2D CE-T1 Spin Echo^a,b^ or DIXON^a^	Oblique axial	≤ 2 mm	≤ 0.3 × 0.3 mm	Choroid, Sclera Optic nerve
2D CE-T1 TSE^a^ or DIXON^a^	Oblique sagittal	≤ 2 mm	≤ 0.3 × 0.3 mm	Choroid, Sclera Optic nerve
DWI (orbits)	Axial	≤ 4 mm		(Active) tumor Optic nerve
3D CE-T1 TSE (brain)	Sagittal	1 mm		Pineal gland/suprasellar region Brain anomalies Leptomeningeal lesions Preauricular and submandibular lymph nodes
If extensive optic nerve invasion or embryonal midline tumor				
CE-T1 TSE spinal axis	Sagittal	≤ 3 mm		Leptomeninges
Optional				
2D T2 TSE^a^(orbits)	Oblique axial or sagittal	≤ 2 mm		Intralesional calcifications Signal intensity of the optic nerve
DWI (brain)	Axial	≤ 4 mm		
T2 TSE or FLAIR (brain)	Axial	≤ 4 mm		
SWI	Axial			Intralesional calcifications
TOF	Axial			Origin and course of the ophthalmic artery

^a^ For detailed planning instructions on axial and sagittal oblique sequences, refer to Fig. 2. These instructions utilize the 3D high-resolution heavily T2 WI to plan orientation along the optic nerve

^b^ The same T1WI sequences should be acquired before and after contrast to enable the calculation of subtraction images

## Timing of imaging

Early detection and prompt treatment are crucial for retinoblastoma. High-resolution contrast-enhanced orbital and brain MRI is recommended to be performed within the first 2 weeks of diagnosis, with initial screening for midline embryonal tumor.

Imaging during eye-conserving treatment or post-treatment is not routinely performed and is reserved for cases where monitoring with fundoscopy is limited, particularly when tumors remain uncontrolled or are located near the optic disc. Given the primary concern of tumor extension into the optic nerve and the potential for leptomeningeal spread, at minimum, the essential sequences of the MRI protocol should be included in follow-up imaging. While some groups recommend follow-up MRI after enucleation in cases with metastatic risk factors [27], no general consensus exists.

Furthermore, no standardized screening protocol currently exists for patients with a germline *RB1* mutation [37–39].

## Imaging analysis and key points for reporting

### Orbit

#### Ocular globe

The eye size in retinoblastoma may vary, appearing larger, normal, or smaller than the normal eye [40]. Increased intraocular pressure may cause buphthalmos (i.e., enlarged eye volume), with additional MRI features such as deformed globe contours and reduced anterior chamber depth. Elevated intraocular pressure is often associated with high-risk histopathologic features [41]. Severe buphthalmos might also increase the risk of surgical complications (i.e., eye rupture and orbital seeding). In retinoblastoma cases with orbital cellulitis, an enlarged globe is frequently observed [9].

Tumor focality significantly influences treatment decisions, as multifocal or bilateral tumors are associated with germline mutations, making conservative treatments the preferred approach. High-spatial-resolution 3D heavily T2-weighted sequences allow precise evaluation of tumor number, size and location.

Previous studies have identified a correlation between tumor size and its extent [10, 11, 33, 42]. Larger intraocular tumors (≥ 16 mm) strongly associate with postlaminar optic nerve invasion (PLONI) and moderately associate with massive choroidal invasion [10, 11].

The intraocular tumor location is assessed relative to the ocular equator (anterior or posterior) and quadrants (nasal or temporal, superior or inferior), the optic disc and macula. In younger patients, retinoblastoma typically originates in the posterior pole of the eye, whereas in older patients, it tends to arise in more anterior or peripheral parts of the retina [43, 44]. Proximity to the optic disc is critical due to the risk of spread through the optic nerve and tumors near the macula are associated with significant vision loss.

Retinoblastoma growth patterns include endophytic, exophytic, and diffuse infiltrative types. Endophytic tumors arise from the inner layers of the retina and grow into the vitreous. Small clusters of tumor cells may detach and cause vitreous seeding, which worsens the prognosis for eye preservation. Exophytic tumors begin in the outer retinal layers, expand into the subretinal space, and cause retinal detachment and possible subretinal seeding. On MRI, the retina is only visible if it is detached, appearing as a fine hypointense line on T2-weighted images. Subtotal retinal detachment connects with the normally attached retina, and total detachment has a characteristic V-shape, with the tip of the “V” centered on the papilla. Vitreous and subretinal seeds may appear as small hypointense lesions on heavily T2-weighted 3D sequences. However, ophthalmoscopy remains the gold standard for detecting these lesions. Mixed growth patterns, combining endophytic and exophytic features, are more common than isolated patterns [32]. Diffuse infiltrating retinoblastoma presents as a placoid mass, infiltrating the retina and vitreous without forming a distinct mass. This pattern may mimic inflammatory or hemorrhagic conditions and resemble other, more innocuous diseases [45].

Predicting high-risk features in retinoblastoma is pivotal in selecting appropriate treatments. Risk factors for local recurrence and metastasis include massive choroidal invasion, scleral and extrascleral invasion, PLONI, and tumor invasion into the trabecular meshwork and Schlemm’s canal in the anterior chamber angle.

**Fig. 3:**
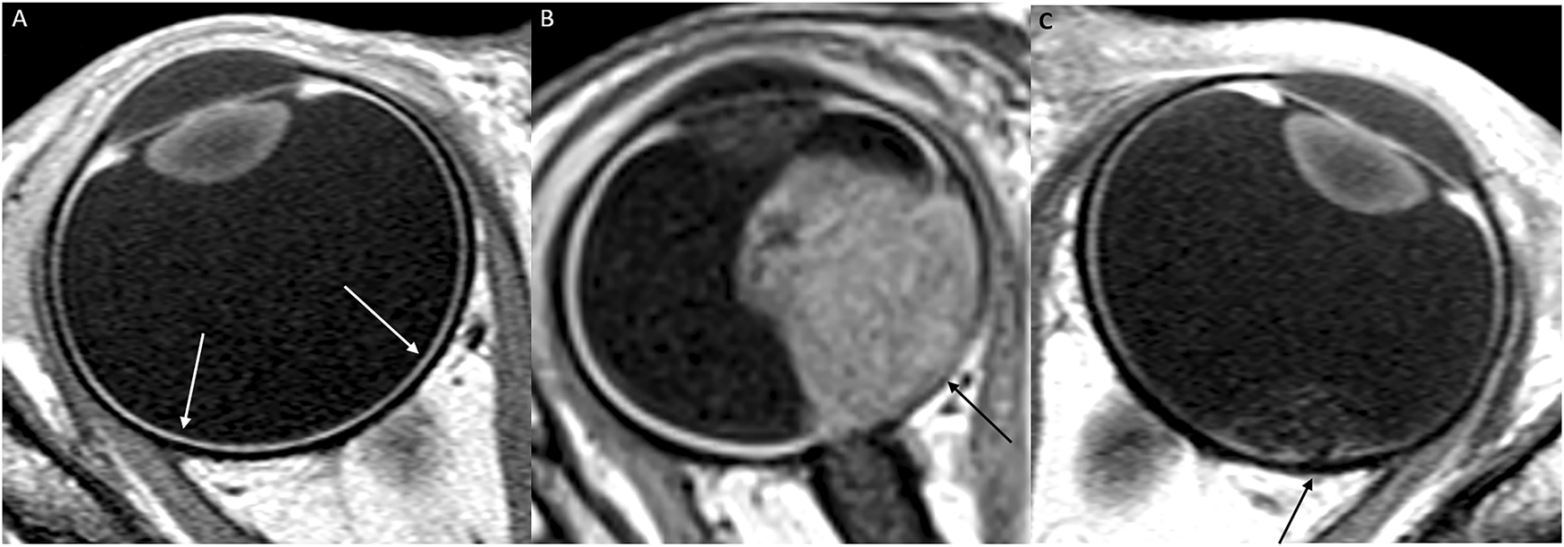
Choroid assessment: **A** Axial contrast-enhanced T1-WI demonstrating a normal, uniformly enhancing choroid (arrows). **B** Axial contrast-enhanced T1-WI showing a large endophytic mass, accompanied by a distinct choroidal enhancement defect (black arrow), consistent with massive choroidal invasion. **C** Axial contrast-enhanced T1-WI revealing a smaller endophytic mass in the macular region with a focal choroidal enhancement defect (black arrow), also indicative of massive choroidal invasion. Note that this is an image after systemic chemotherapy

Choroidal invasion is classified as “massive” when choroidal infiltration exceeds 3 mm in its largest diameter on microscopic histopathologic analysis [46], which increases the risk of hematogenous spread and mortality. Normally, the choroid appears as a thin, regular enhancement on contrast-enhanced MR images. Enhancement defects or localized thickening of the choroid raise suspicion for profound choroidal invasion (Fig. 3) [42]. Partial volume effects at the globe’s curvature may limit interpretation, making analysis of contrast-enhanced high-resolution MR images in at least two planes essential for reliable assessment [12]. However, subtle choroidal invasion on MRI remains challenging to detect. Notably, even small tumors may present a risk for massive choroidal invasion [11].

**Fig. 4:**
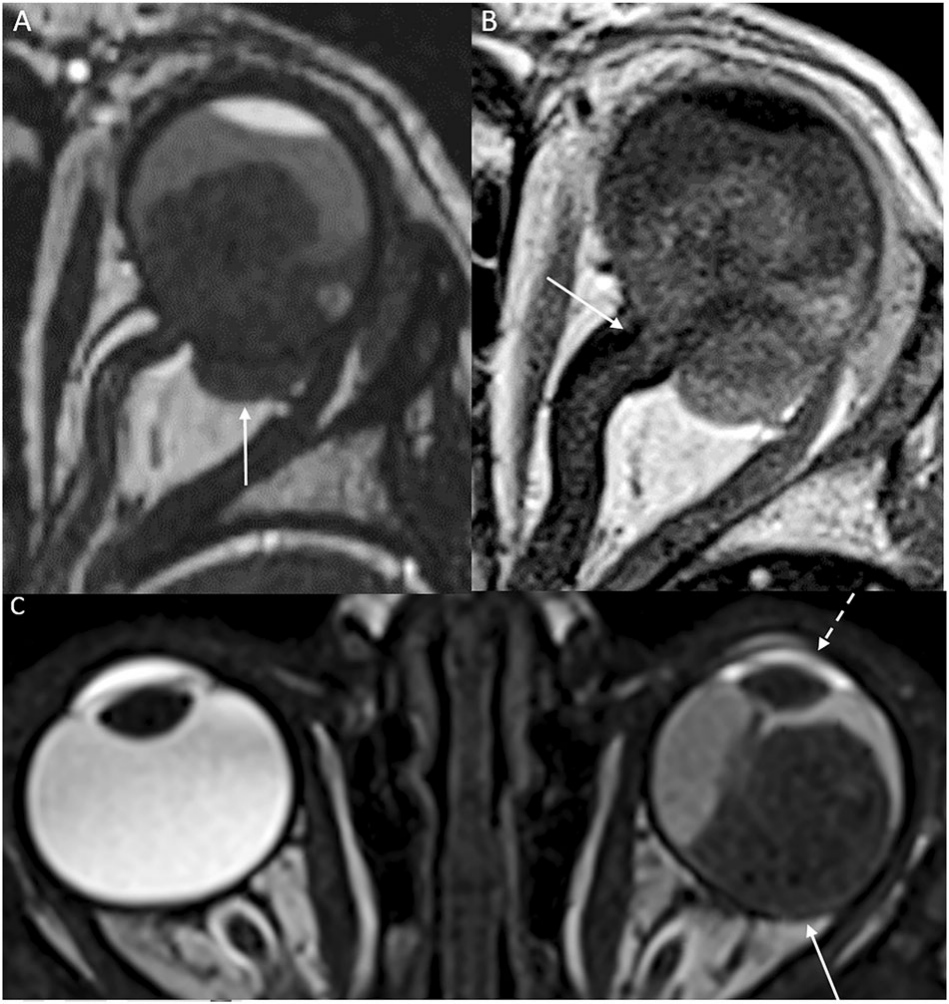
Scleral assessment: **A** Axial T2-weighted MR image showing an interruption of the hypointense sclera with evidence of extrascleral extension (arrow). **B** Axial contrast-enhanced T1-WI confirming the interruption and extension. This tumoral extension is in contact with the lateral border of the meningeal sheath of the optic nerve, the medial side of the lateral rectus muscle, and the orbital fat. Additionally, note the enhancement of the postlaminar optic nerve (arrow). **C** Axial heavily T2-weighted image of a 3-month-old infant demonstrating an exophytic tumor in the left eye, causing enlargement of the globe (buphthalmos) with focal distortion of the posterior temporal sclera (arrow). No evidence of scleral extension was found on histopathological examination after enucleation. Additionally, note the shallow anterior chamber, compared to the normal right eye (dashed arrow)

Scleral invasion appears on MRI as an interruption in the normal sclera that is hypointense on both T1- and T2-weighted images. Extrascleral invasion is defined as tumor spread beyond the globe, infiltrating the orbital fat (Fig. 4). This form of extension is rare in high-income countries but may occur in children with delayed diagnosis. As with choroidal invasion assessment, multi-plane image analysis is important to avoid misinterpretation from partial volume effects. Studies report good sensitivity and specificity for detecting scleral invasion and/or extrascleral spread [14]. Distortion of the sclera by the tumor, especially in newborns, may cause focal thinning, which should not be interpreted as scleral extension if the sclera remains continuous and no extrascleral abnormalities are observed.

Increased anterior chamber enhancement can result from hyperemia, uveitis or iris neovascularization. De Graaf et al [47] found a strong correlation between anterior eye segment enhancement and tumor volume. Additional studies have identified significant associations between anterior eye segment enhancement and optic nerve invasion [13, 15, 47, 48], though correlation with choroidal invasion is less consistent.

A shallow anterior chamber may be observed in eyes with elevated intraocular pressure. Chawla et al [13] found a significant association between a shallow anterior chamber and tumor invasion of the iris.

**Fig. 5:**
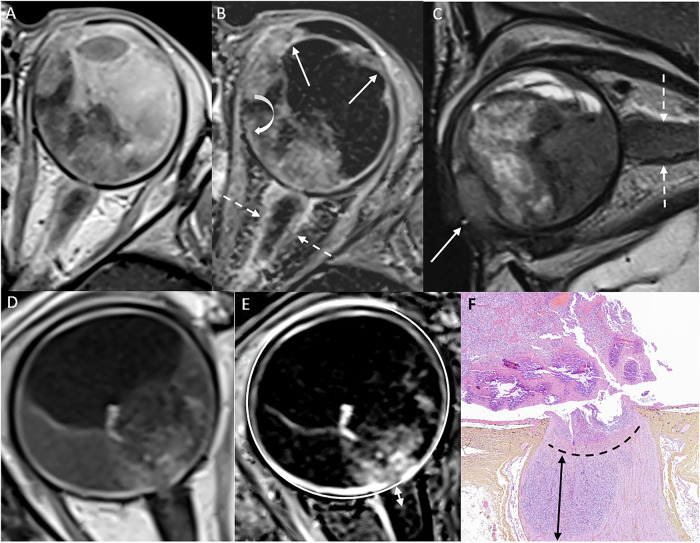
Anterior segment and optic nerve invasion: **A** Axial contrast-enhanced T1-weighted image and (**B**) axial contrast-enhanced subtraction T1-weighted image showing anterior intraocular extension with infiltration of the ciliary body (arrows), massive choroidal invasion on the nasal side (curved arrow) and extensive enhancement along the optic nerve (dashed arrows). **C** Sagittal T2-weighted image showing the marked anterior tumoral infiltration with extraocular extension (arrow) and enlargement and hyperintense signal of the optic nerve (dashed arrows). **D** Axial contrast-enhanced T1-weighted image and **E** axial subtraction contrast-enhanced T1-WI showing the intersection of the optic nerve with a circle drawn at the outer border of the enhancing choroid, which delimits the location of the lamina. The length of the postlaminar enhancement is measured (double-headed arrow) to estimate the postlaminar invasion of the optic nerve. **F** Histopathological correlation reveals the presence of tumor cells (double-headed arrow) located posterior to the lamina cribrosa (dashed line)

Large tumors in the posterior segment may extend forward, invading the ciliary body. Less commonly, retinoblastoma is located anteriorly, usually in older children or MYCN-amplified retinoblastoma cases [30]. Ciliary body invasion on MRI, compared with histopathology, shows sensitivities of 71–100% and specificities of 65–100% in a limited number of studies [14]. Contiguous tumor infiltration and focal areas of reduced contrast enhancement in the ciliary body should alert the radiologist (Fig. 5). However, minimal ciliary body invasion remains challenging to confirm on MRI due to spatial resolution limitations.

#### Optic nerve

Optic nerve assessment is essential for accurate staging in retinoblastoma and plays a crucial role in guiding treatment strategies. This is the main reason for performing MRI, as optic nerve invasion cannot be detected through clinical examination. First, optic nerve invasion may contraindicate primary enucleation due to the risk of cutting an invaded segment of the optic nerve, potentially leading to orbital tumor seeding [49]. Additionally, PLONI is a known prognostic factor, associated with increased risks of recurrence and metastasis [50]. Since fewer than 30% of retinoblastoma cases are treated with enucleation, histopathological diagnosis, which remains the gold standard for identifying high-risk features in retinoblastoma, is available for only a limited number of patients. MRI, therefore, serves as the primary method for evaluating optic nerve invasion in most patients, supporting eye-conserving treatments.

A significantly enlarged optic nerve is the most reliable indicator of tumor invasion, often accompanied by abnormal optic nerve enhancement (Fig. 5). High-resolution T2 and contrast-enhanced T1-weighted MRI are crucial for evaluating the optic nerve, with sensitivity and specificity rates of 60% and 95–100%, respectively [10, 14, 33]. De Bloeme et al identified a threshold of ≥ 2.215 mm for the optic nerve diameter, just posterior to the lamina cribrosa, measured on the 3D high-resolution T2 sequence, with an 84% sensitivity and 83% specificity for detecting PLONI [16]. However, limited nerve invasion may present with a normal optic nerve size, making enhancement evaluation essential. Fat suppression and subtraction techniques can help clarify or rule out optic nerve enhancement. False negative results are rare, and a normal optic nerve size and signal on high-resolution T2-weighted images, with minimal (< 3 mm) or no enhancement, are reliable criteria to rule out advanced optic nerve invasion. False positives are more common, and radiologists should be aware of conditions that might mimic optic nerve invasion, such as the “double dots” sign (i.e., two enhancing foci near central retinal vessels), normal central retinal vessel enhancement, and posterior bulging of the lamina cribrosa due to elevated intraocular pressure [10]. Inflammatory enhancement of the optic nerve in cases of retinoblastoma with orbital cellulitis can be distinguished from tumor invasion on MRI [9]. Indirect indicators of optic nerve invasion include tumor size and anterior segment enhancement. Recently, radiomic analysis has been proposed to improve predictive accuracy for PLONI [17].

To measure the length of the postlaminar optic nerve enhancement, the location of the lamina cribrosa is estimated at the junction of the optic disc and a circle drawn along the choroid’s outer border (Fig. 5). Extensive optic nerve invasion occurs more frequently in low-income countries due to delayed diagnoses. Evaluating the different segments of the optic nerve (orbital, cisternal/prechiasmatic and chiasmic) is crucial for determining whether surgery is feasible and for avoiding the cutting of a potentially invaded segment of the optic nerve. The width of tumor invasion of the optic nerve, or its proximity to the leptomeninges, may be associated with an increased risk of metastatic spread to the cerebrospinal fluid [18].

### Central nervous system

#### Neuraxis

If there is extensive optic nerve invasion or extension along the meningeal sheath, it is imperative to complete imaging of the entire neuraxis (prior to lumbar puncture), including the dural sac, to assess leptomeningeal disease.

#### Midline embryonal tumor

**Fig. 6:**
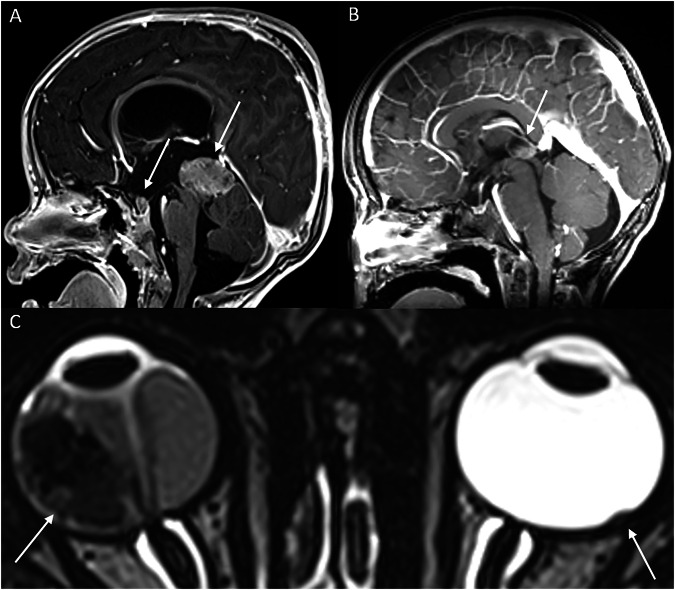
Midline embryonal tumors and trilateral retinoblastoma: **A** Sagittal contrast-enhanced T1-weighted image of a 2-year-old boy with a history of bilateral retinoblastoma since birth, showing midline embryonal tumors in both the suprasellar and the pineal regions (arrows). **B** Sagittal contrast-enhanced T1-weighted image demonstrating a pinealoblastoma (arrow). **C** Axial heavily T2-weighted image from the same patient revealing bilateral retinoblastoma (arrows)

About 3.5–3.8% of children with hereditary retinoblastoma are at risk of developing CNS embryonal tumors, histologically similar to retinoblastoma, in the pineal region or, less frequently, in the suprasellar region (Fig. 6). This presentation, known as “trilateral” retinoblastoma, carries a significantly higher mortality risk [19]. Early detection of asymptomatic pineal trilateral retinoblastoma improves survival outcomes compared to symptomatic cases of pinealoblastoma [20]. Thus, baseline brain MRI is mandatory at diagnosis.

An enlarged solid or cystic pineal gland or irregular thickening of the cyst wall (> 2 mm) is suspicious for pinealoblastoma. When a midline embryonal tumor is diagnosed, complete neuraxis imaging is required to assess for spinal leptomeningeal disease. Galluzzi et al [21] and Sirin et al [22] have proposed age-based normal values for the size and morphology of the solid and cystic pineal gland in children, along with management recommendations. If the baseline MRI shows a solid or harmoniously cystic pineal gland within the 99% prediction interval, only clinical follow-up is advised. Conversely, a cystic pineal gland with atypical or irregular morphology warrants at least one follow-up MRI at three months [23]. If MRI findings reveal suspicious changes, growth or obvious pinealoblastoma, appropriate treatment should be initiated. Freire et al proposed normal ADC values for the pineal gland in children under 4 years of age. The mean ADC value of the normal pineal gland tends to be higher than that observed in patients with trilateral retinoblastoma [51].

The incidence of metachronous pineal trilateral retinoblastoma in hereditary retinoblastoma patients is estimated at less than 2% [24]. Although brain MRI before the age of 12 months in these patients often misses pinealoblastomas, and retinoblastomas are typically diagnosed before this age [25], there is currently no consensus on systematic screening for pinealoblastoma in hereditary retinoblastoma patients [23].

#### Brain

The 13q deletion syndrome is associated with retinoblastoma and other structural brain abnormalities, such as corpus callosum anomalies and Dandy-Walker malformation [52]. Children with this syndrome may also exhibit facial dysmorphism (e.g., anteverted ear lobes, a high and broad forehead, prominent philtrum), severe intellectual disability and/or motor impairment [53].

### Non-central nervous system lesions

Preauricular and submandibular lymph nodes are the first relay for lymphatic drainage in retinoblastoma. Lymph node invasion is rare at diagnosis, typically occurring in patients with extensive anterior eye segment or orbital invasion, or in cases of recurrence.

Distant non-central nervous system and systemic metastases are rare in retinoblastoma patients in developed countries. The typical metastatic sites include the bone marrow, and less frequently the lungs and liver.

A checklist for structured MRI reporting is provided in Table 3.

**Table 3:**
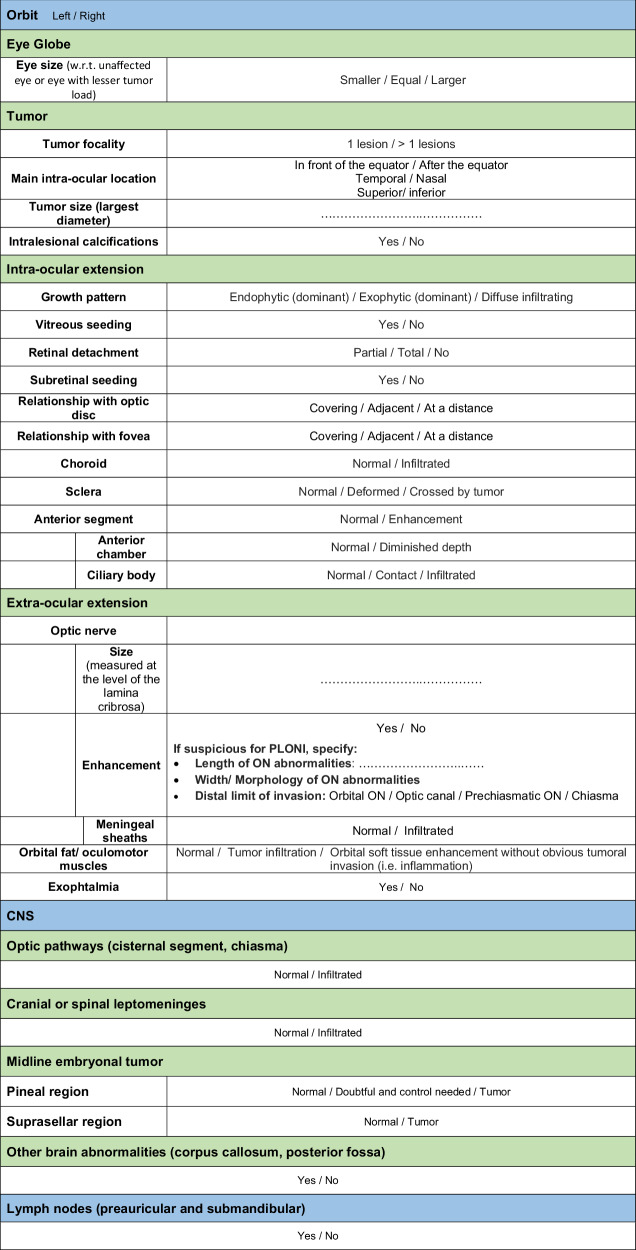
Checklist for MRI reporting

## Differential diagnosis

Several conditions can mimic retinoblastoma, with Coats’ disease and persistent fetal vasculature being the most common. MR imaging features can help differentiate these conditions: retinoblastoma often shows larger eye size, vitreous seeding, and V-shaped retinal detachment, while pseudoretinoblastoma shows smaller eye size, ciliary/lens deformations, optic nerve atrophy, a central stalk between optic disc and lens, Y-shaped retinal detachment, and absence of calcifications. Additionally, features like intraretinal macrocysts, contrast enhancement outside the solid lesion, and subfoveal nodules can indicate pseudoretinoblastoma [4]. Zhang et al recently showed the benefit of ADC measurements for differentiating retinoblastoma from Coats [28].

Ocular toxocariasis, typically affecting children around 8 years old, can mimic retinoblastoma by presenting as leukocoria due to pseudo-tumoral retinal or vitreous lesions.

Intraocular medulloepithelioma, though rarer, is the second most common intraocular tumor in children and can be differentiated from retinoblastoma by its mixed solid and cystic nature, absence of calcifications and higher ADC values [54, 55]. In 5% of cases, an association with DICER1 syndrome exists [56].

## Future developments

Recent research on the molecular basis of retinoblastoma [57, 58] highlights the importance of non-invasive diagnostic methods, as biopsy of an ocular tumor carries significant risks, potentially compromising vision and vital prognosis. Radiogenomic biomarkers [29, 30] and cfDNA analysis from aqueous humor [59] offer promising approaches for identifying molecular subtypes, thus supporting personalized treatment.

## Abbreviations

PLONI: Postlaminar optic nerve invasion
SE: Spin echo
