# Correction: Practical guidelines on imaging of retinoblastoma: a 2025 update on behalf of the European Retinoblastoma Imaging Collaboration and the European Retinoblastoma Group

**DOI:** 10.1007/s00330-025-12005-1

**Published:** 2025-09-11

**Authors:** Liesbeth Cardoen, Selma Sirin, Paolo Galluzzi, Marcus C. de Jong, Meriam Koob, Sophia Göricke, Christiaan M. de Bloeme, Maaike Moor, Isabelle Aerts, Alexandre Matet, Annette C. Moll, François Doz, Pim de Graaf, Hervé J. Brisse

**Affiliations:** 1 Imaging Department, Institut Curie, Université Paris Sciences et Lettres, Paris, France; 2 Department of Diagnostic Imaging, University Children’s Hospital Zürich, Zürich, Switzerland; 3 Neuroradiology Unit Azienda Ospedaliera Universitaria Santa Maria alle Scotte, Siena, Italy; 4 Department of Radiology and Nuclear Medicine, Amsterdam UMC location Vrije Universiteit Amsterdam, Cancer Center Amsterdam, Imaging and Biomarkers, Amsterdam, The Netherlands; 5 Department of Radiology, Lausanne University Hospital (CHUV) and University of Lausanne (UNIL), Lausanne, Switzerland; 6 Institute of Diagnostic and Interventional Radiology and Neuroradiology, University Hospital Essen, Essen, Germany; 7 SIREDO Oncology Center Care, Innovation and Research for Children, Adolescents and Young Adults with Cancer, Institut Curie, Paris, France; 8 Department of Ophthalmology, Institut Curie, Université Paris Cité, Paris, France; 9 Department of Ophthalmology, Amsterdam UMC location Vrije Universiteit Amsterdam, Cancer Center Amsterdam, Cancer Treatment and Quality of Life, Amsterdam, The Netherlands

**Correction to: European Radiology**

10.1007/s00330-025-11853-1 published online 06 Aug 2025.

In this article the author’s name Paolo Galluzzi was incorrectly written as Paolo Galuzzi.

The original article has been corrected.

